# Constitutive and Treatment-Induced CXCL8-Signalling Selectively Modulates the Efficacy of Anti-Metabolite Therapeutics in Metastatic Prostate Cancer

**DOI:** 10.1371/journal.pone.0036545

**Published:** 2012-05-09

**Authors:** Catherine Wilson, Pamela J. Maxwell, Daniel B. Longley, Richard H. Wilson, Patrick G. Johnston, David J. J. Waugh

**Affiliations:** Centre for Cancer Research and Cell Biology, Queen's University Belfast, Belfast, Northern Ireland; The Chinese University of Hong Kong, Hong Kong

## Abstract

**Background:**

The current study was undertaken to characterize the effect of anti-metabolites on inducing CXCL8 signaling and determining whether the constitutive and/or drug-induced CXCL8 signaling in metastatic prostate cancer (CaP) cells modulates their sensitivity to this class of agent.

**Methods:**

The response of metastatic CaP cells to 5-Fluorouracil (5-FU), Pemetrexed or Tomudex was determined using cell count assays, flow cytometry and PARP cleavage analysis. Quantitative-PCR, ELISA and immunoblots were employed to determine effects of drugs or CXCL8 administration on target gene/protein expression.

**Results:**

Administration of 5-FU but not pemetrexed potentiated CXCL8 secretion and increased CXCR1 and CXCR2 gene expression in metastatic PC3 cells. Consistent with this, the inhibition of CXCL8 signaling using a CXCR2 antagonist, AZ10397767, increased the cytotoxicity of 5-FU by 4-fold (P<0.001), and increased 5-FU-induced apoptosis in PC3 cells (P<0.01). In contrast, while administration of AZ10397767 had no effect on the sensitivity of pemetrexed, the CXCR2 antagonist exerted the greatest effect in increasing the sensitivity of PC3 cells to Tomudex, a directed thymidylate synthase (TS) inhibitor. Subsequent experiments confirmed that administration of recombinant human CXCL8 increased TS expression, a response mediated in part by the CXCR2 receptor. Moreover, siRNA-mediated knockdown of the CXCL8-target gene Bcl-2 increased the sensitivity of PC3 cells to 5-FU.

**Conclusions:**

CXCL8 signaling provides a selective resistance of metastatic prostate cancer cells to specific anti-metabolites by promoting a target-associated resistance, in addition to underpinning an evasion of treatment-induced apoptosis.

## Introduction

Treatment of advanced castrate-resistant metastatic prostate cancer (CRPC) remains a significant clinical unmet need. The recent approval of abiraterone-acetate as a second-line treatment in CRPC is a major advance, however, this agent which targets the androgen synthesis pathway provides only a limited improvement in overall survival and only patients with a good performance status benefit from its provision [1], [2]. Many patients will not meet the preferential performance status that is optimal for response to abiraterone. Moreover, many tumours will be resistant to abiraterone in second-line treatment. Consequently, this mandates that efforts to expand the armoury of agents available to clinicians treating CRPC is not relaxed. Such efforts should not be restricted solely to the discovery of new agents but should exploit our continuing knowledge of the disease biology to define mechanisms of resistance and identify novel agents which can be exploited to improve the efficacy of existing chemotherapy agents.

Conventional chemotherapy has been largely ineffective in the treatment of CRPC; docetaxel remains the sole chemotherapy agent to have obtained approval for CRPC, on the basis of a limited improvement in overall survival [3]. The anti-metabolite 5-FU has been a mainstay of solid tumor chemotherapy for over five decades; upon entering the cell, 5-FU is converted to three active metabolites, fluorouridine triphosphate (FUTP), flurodeoxyuridine triphosphate (FdUTP) and flurodeoxyuridine monophosphate (FdUMP), which are mis-incorporated into RNA, promote DNA damage, or contribute to the inhibition of the enzyme thymidylate synthase, respectively [4]. Recent phase II studies of 5-FU and its oral analog capecitabine have shown them to be safe in second-line treatment for metastatic CRPC, with modest responses observed when administered in combination with docetaxel or oxaliplatin [5], [6]. Although these clearly remain sub-optimal responses, the capacity to target rapidly proliferating CaP cells by inducing an S-phase block and subsequently apoptosis induction remains an attractive therapeutic scenario, especially in tumors that have become refractory to anti-androgen therapy.

Prostate cancer cells are subject to a pronounced autocrine CXCL8 signaling stimulus, which increases with stage of disease and is maximal in castrate-resistant disease [7], [8]. The magnitude of CXCL8 signaling is potentiated by environmental factors such as hypoxia [9] and chemically-induced stresses including exposure to chemotherapy agents [10], [11], which concurrently regulate expression of the ligand and the receptors through which it mediates its biological effects, i.e. CXCR1 and CXCR2. CXCL8 promotes progression to castrate-resistance through ligand-independent activation of the androgen receptor (AR) [12], [13] and induces the proliferation of metastatic prostate cancer cells [7], [14] (). Furthermore, we have shown that stress-induced CXCL8 signaling attenuates the sensitivity of prostate cancer cells to undergo apoptosis in response to DNA-damaging agents [9], [10], Hsp90-directed inhibitors [15], death receptor agonists (TRAIL) [11] and AR-targeted therapeutics such as bicalutimide (Casodex) [13].

The aim of this study was to determine how intrinsic and/or treatment-induced CXCL8 signaling modulates the response of CRPC cells to anti-metabolites.

## Materials and Methods

### Chemical and Reagents

All chemicals were source from Sigma-Aldrich (St. Louis, MO) unless otherwise stated. 5-FU was obtained from Bridgewater Chemotherapy Suite, Belfast City Hospital. Pemetrexed was a kind gift from Dr Dean Fennell (CCRCB, QUB, Belfast, UK). AZ10397767 was kindly provided by Dr. Simon T. Barry and Dr. David Blakey (AstraZeneca, Alderley Park, Cheshire, UK).

### Cell Culture

PC3 prostate cancer cells were cultured as previously described [10]. In subsequent experiments investigating CXCL8 signaling, PC3 cells were incubated in serum-free RMPI 1640 medium for 16 h prior to exposure to 3 nM recombinant human (rh)-CXCL8 (Peprotech, London, UK).

### Cytotoxic agents

5-FU and pemetrexed were stored at 4°C as 10 mM stock solutions. Serial dilutions were made up in sterile injection water and drugs were added directly to cells to yield the desired concentration of the cytotoxic agent. In the case of 5-FU treatment, cells were maintained and treated in medium containing dialysed FCS.

### siRNA Transfections

siRNA transfections were carried out as previously described [11]. Cells (5×10^5^) were washed twice in sterile PBS and then incubated with a transfection mixture comprising optimem medium, oligofectamine (Invitrogen, Paisley, UK), and the specific oligonucleotide (Bcl-2 200 nM; Dharmacon Inc) for 48 h at 37°C. A non-targeting sequence was included in these experiments at the same concentration as the siRNA sequence used.

### Cell Count Analysis

Cells (1×10^5^ cells/well) were allowed to adhere overnight. Media was replaced with fresh RPMI 1640 medium and treated with a range of concentrations of 5-FU or pemetrexed in the absence or presence of AZ10397767 (20 nM). Cells were incubated at 37°C in a humidified 5% CO_2_ atmosphere for 72 h, then trypsinized and counted using a Coulter® Z^TM^ Series particle count and size analyzer (Beckman Coulter, Miami, FL) as previously described [10]. Cell numbers were normalized to control values and statistical analyses of the data performed using GraphPad PRISM 3.0 software.

### Flow Cytometry

Cell-cycle analysis was evaluated by propidium iodide staining of cells as previously described [10].

### Immunoblotting

Whole cell lysates were prepared, resolved and blotted as described previously [7]. Membranes were washed in Tris-buffered saline/ 0.1% Tween-20 (TBS-T) then blocked for 1h at room temperature in 5% bovine serum albumin/TBS-T (BSA/TBS-T). Membranes were probed with primary antibodies to Bcl2 (Santa Cruz Biotechnology Inc., Santa Cruz, CA), TS (Rockland Immunochemicals Inc., Gilbertsville, PA) or PARP (eBiosciences Inc., San Diego, CA, USA). The membranes were washed three times in TBS-T and then incubated with a horse-radish peroxidase (HRP)-labelled secondary antibody (GE Healthcare UK Ltd, Buckinghamshire, UK). Following three washes in TBS-T the bands were detected using enhanced chemiluminescence (ECL plus reagents, Amersham Biosciences). Membranes were re-probed with GAPDH antibody to ensure equal loading (Biogenesis, Dorset, UK).

### Quantitative real time PCR (qPCR)

RNA was harvested as previously described [9]. For real-time PCR, 1μg of total RNA was oligo(dT) reverse transcribed using MMLV-RT (Invitrogen, Paisley, UK) according to the manufacturer's instructions. 50 ng cDNA was mixed with primers (2 nM), sterile water and SYBR Green PCR mastermix (Roche Diagnostics, Sussex, UK). The primer sequences were as previously reported [9]. Real-time PCR was carried out in a 96-well plate using an LC480 light cycler instrument (Roche Diagnostics). The threshold cycle (C_P_) was calculated for each reaction by the LC480 system. Unknown expression levels were determined using the relative standard curve method and normalised against 18S.

### ELISA

The amount of secreted CXCL8 detected in the medium of prostate cancer cells was determined using an ELISA assay according to the manufacturer's instructions (Pelikine Compact CXCL8 ELISA kit, Beckman Coulter, High Wycombe, UK) as previously described [9]. The CXCL8 concentration in each sample was calculated by comparison to a standard curve generated from a stock vial of CXCL8.

## Results

### 5-FU potentiates chemokine signaling in metastatic CaP cells

**Figure 1:**
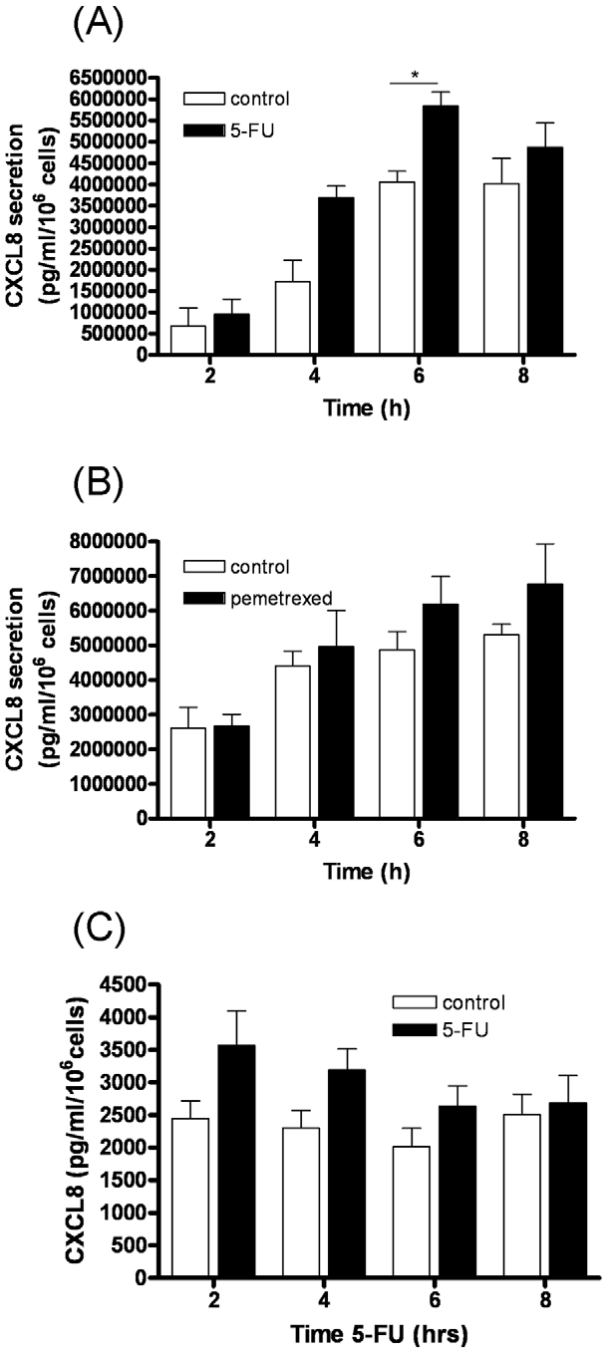
Effects of anti-metabolite agents upon CXCL8 secretion from prostate cancer cells. (**A**) Bar graph plotting the level of CXCL8 secretion following treatment of PC3 cells with 1 µM 5-FU. Values shown are plotted relative to a time-matched control and represent the mean ± S.E.M. of three independent experiments. (**B**) As in A, except that cells were treated with 1 µM pemetrexed. Differences in CXCL8 expression between time-matched control and drug-treated samples were analyzed by a two-tailed Student's t-test; *, p<0.05. (**C**) Bar graph plotting the levels of CXCL8 secretion following treatment of LNCaP cells with 1 µM 5-FU. Values shown are plotted relative to a time-matched control and represent the mean ± S.E.M. of three independent experiments.

The PC3 cell line was originally isolated from a bone metastasis of CaP and therefore is considered to be a clinically-relevant model system to study advanced CaP [16]. Since prior studies have shown chemotherapy agents induce CXCL8 synthesis, our initial experiments focused on characterizing the effects of administering anti-metabolites upon the secretion of this chemokine. In addition to using 5-FU, our initial experiments were also conducted using pemetrexed, a multi-targeted anti-folate which inhibits thymidylate synthase (TS), dihydrofolate reductase (DHFR), and glycinamide ribonucleotide formyltransferase (GARFT), all enzymes involved in the *de novo* biosynthesis of thymidine and purine nucleotides [17]. A specific ELISA was used to quantify CXCL8 secretion from PC3 cells over an 8 h timecourse, comparing time-matched controls from 5-FU-treated or pemetrexed-treated cells. Administration of 5-FU promoted a significant increase in CXCL8 secretion from PC3 cells (P<0.05) (Figure 1A). However, administration of pemetrexed had negligible effects on CXCL8 secretion (Figure 1B). To verify our observations, experiments were repeated in the LNCaP prostate cancer cell line; addition of 1 µM 5-FU also increased the levels of CXCL8 secreted from this cell line (Figure 1C).

**Figure 2:**
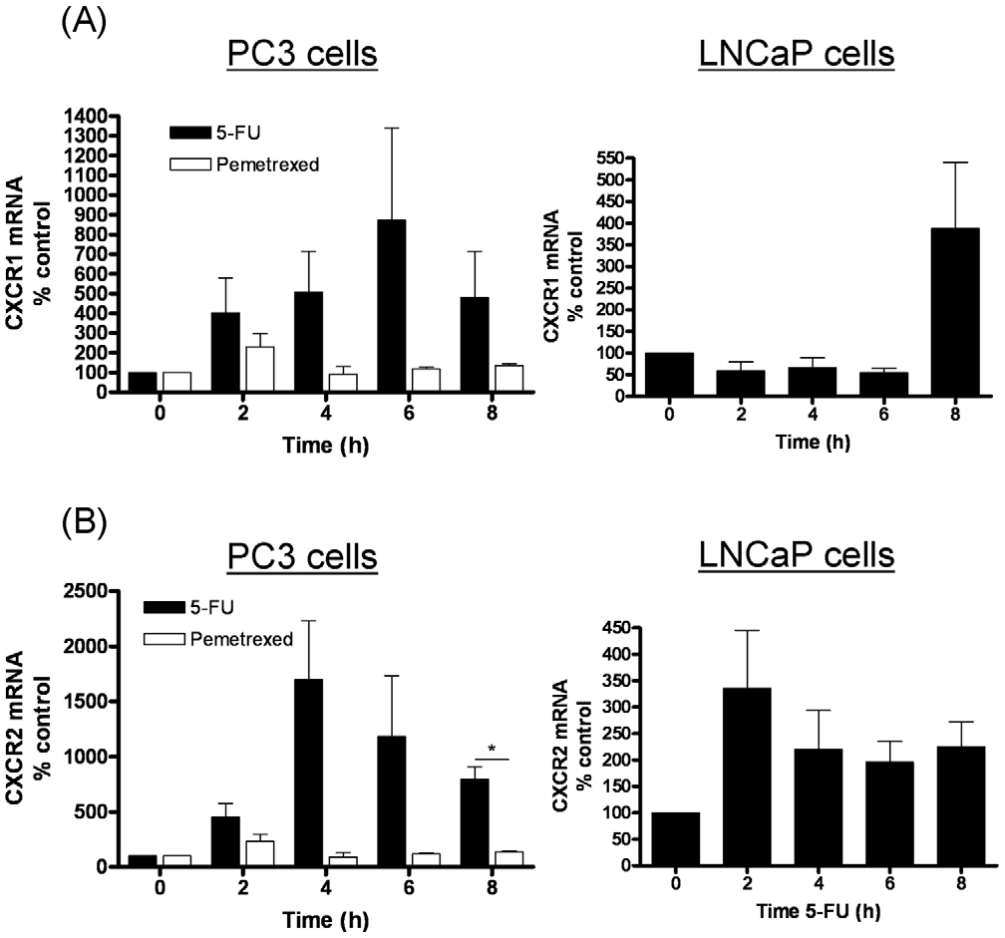
Effects of anti-metabolite agents upon the gene expression of CXCL8 receptors in prostate cancer cells. (**A**) Bar graph presenting the time-dependent change in CXCR1 transcript levels in PC3 cells (left panel) and LNCaP cells (right panel) as detected by qPCR analysis following treatment with 1 µM 5-FU (black bars) or 1 µM Pemetrexed (open bars – PC3 cells only). Expression is presented as a fold-change relative to gene expression levels detected in untreated cells. (**B**) As in A, except that data shows the gene expression level detected for CXCR2. Data shown is the mean ± S.E.M. of four independent experiments. Statistically significant differences were determined using a Students two-tailed t-test (*, p<0.05).

The effects of 5-FU and pemetrexed administration upon expression of the CXCL8 receptors were also studied using quantitative PCR analysis. Administration of 5-FU had pronounced and sustained effects upon the gene expression of both CXCR1 (Figure 2A, left panel) and CXCR2 (Figure 2B, left panel), increasing transcript levels by factors of 8- and 15-fold, respectively. In contrast, administration of pemetrexed had a transient and minimal effect upon the gene expression of either receptor. Further experiments confirmed that 5-FU administration also increased CXCR1 and CXCR2 transcript levels in LNCaP cells (Figure 2A/B, right panels), though the response was markedly more selective for CXCR2 in this cell line.

### Inhibition of CXCL8 signaling potentiates 5-FU efficacy in metastatic CaP cells

**Figure 3:**
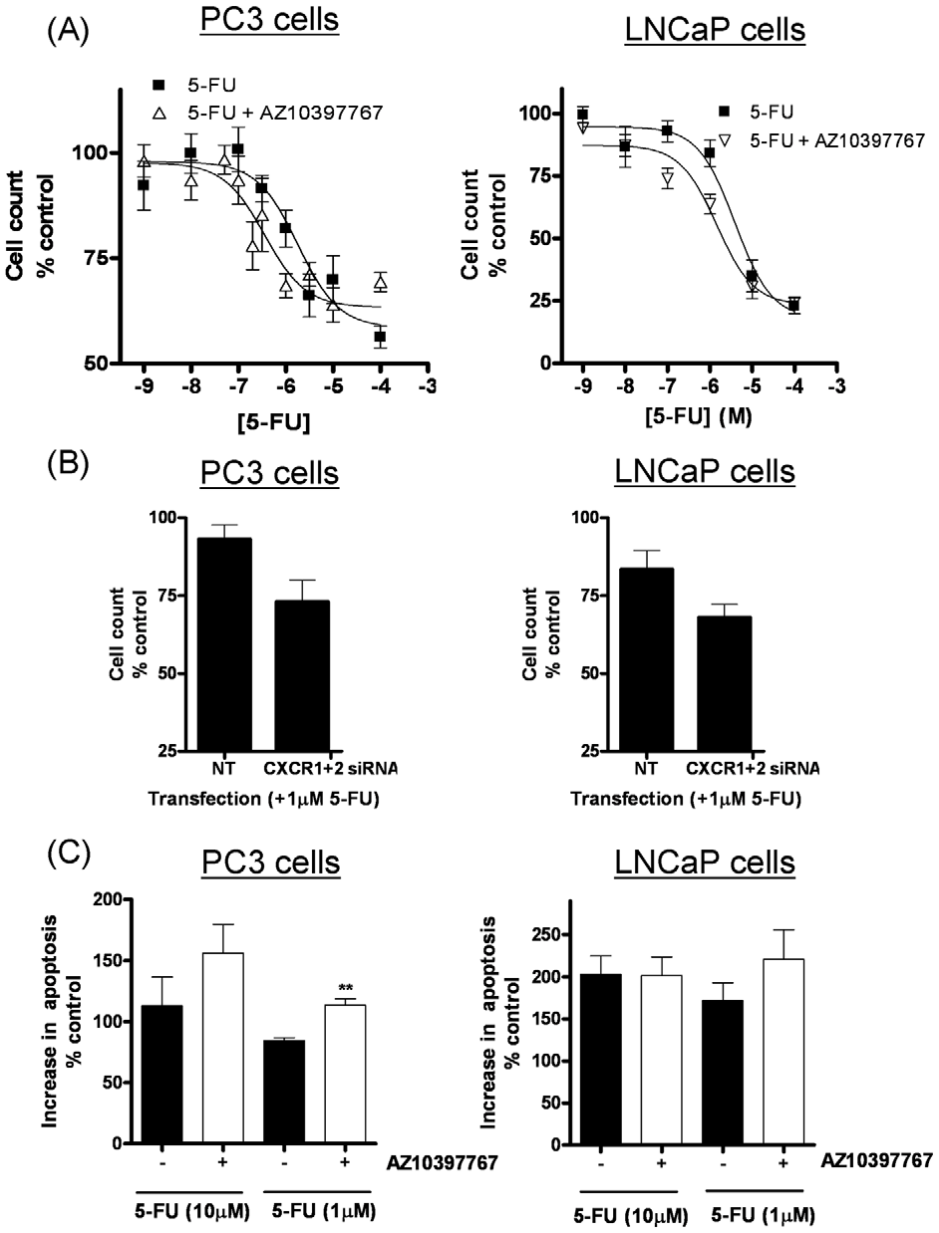
Effect of inhibiting CXCL8/CXCR2 signaling upon the efficacy of anti-metabolites in metastatic prostate cancer cells. (A) Graphs illustrating cell count assay data, determined from the treatment of PC3 cells (left panel) or LNCaP cells (right panel) with increasing concentrations of either 5-FU, in the absence or presence of the CXCR2 receptor antagonist, AZ10397767. AZ10397767 was administered at a final concentration of 20 nM. Cell counts were taken 72 hours post-treatment with the anti-metabolite. Cell count data was analyzed using the non-linear regression function of GraphPad Prism, using a sigmoidal one-site curve fit equation. Data points shown are the mean ± S.E.M. value of four independent experiments. (B) Bar graph illustrating the effect of using siRNA to knockdown CXCR1 and CXCR2 expression upon the cytotoxicity of 1 µM 5-FU in PC3 (left panel) and LNCaP cells (right panel). Data points shown are the mean ± S.E.M. value of four independent experiments. (C) Bar graph presenting the level of apoptotic cells in drug-treated PC3 (left panel) and LNCaP (right panel) cell populations. Data shows the sub G0/G1-cell population determined by flow cytometry analysis following treatment with increasing concentrations of 5-FU, in the absence and presence of the CXCR2 receptor antagonist, AZ10397767.

Cell count assays were employed to characterize the relative cytotoxicity of 5-FU or pemetrexed in PC3 cells and to determine whether drug-induced or intrinsic CXCL8 signaling affected the sensitivity to these agents. Inhibition of CXCL8 signaling was effected using a selective CXCR2 receptor antagonist, AZ10397767. PC3 cells were only sensitive to 5-FU at concentrations exceeding 1 µM (Figure 3A). The addition of AZ10397767, used at a final concentration of 20 nM in order to exercise its selectivity for the CXCR2 receptor increased the potency of 5-FU-induced cytotoxicity in PC3 cells. Non-linear regression analysis of the data confirmed that the inhibition of CXCR2 signaling enhanced the potency of 5-FU by 3.8-fold, increasing the calculated IC_30_ value from 4.2 µM to 1.1 µM (n = 4). Further analysis of the data determined that the addition of AZ10397767 to 5-FU was synergistic by a calculated RI value of 1.2, while ANOVA analysis confirmed a strong synergistic interaction between 5-FU and AZ10397767 (P<0.0001). Similarly, the administration of AZ10397767 increased the sensitivity of LNCaP cells to 5-FU. Repression of CXCR2 signaling had no effect in increasing the maximal response of 5-FU therapy in either cell line. In contrast, the administration of the CXCR2 antagonist did not increase the potency or the maximal response of pemetrexed in the PC3 cells (Supplementary Figure S1). The potency of pemetrexed in these assays was calculated as 0.27 µM.

As an additional validation of our results obtained with the CXCR2 antagonist, we employed a siRNA-strategy to repress receptor expression. Despite using commercial sequences claiming to be selective for CXCR1 or CXCR2 knockdown, subsequent RT-PCR analysis determined significant down-regulation of the reciprocal receptor in the PC3 and LNCaP cells. Irrespective, down-regulation of CXCR1 and CXCR2 expression resulted in a greater loss of PC3 and LNCaP cell viability in the presence of 1 µM 5-FU (Figure 3B).

Flow cytometry analysis confirmed that the inhibition of CXCR2 signaling potentiated 5-FU-induced apoptosis in both PC3 and LNCaP cells. Relative to untreated control cells, an increase in the concentration of 5-FU from 1 µM to 10 µM resulted in an increased accumulation of cells in the S phase of the cell cycle (Supplementary Figure S2) and the detection of an increased percentage of cells in the sub G0/G1 phase (Figure 3C). Addition of AZ10397767 alone to PC3 cells had no observed effect on any phase of the cell cycle at the 72 h timepoint. However when combined with 1 µM 5-FU, AZ10397767 decreased the accumulation of the cells arrested in the S phase of the cell cycle while concurrently increasing the level of apoptotic cells observed (p<0.0045) (Supplementary Figure S2).

### CXCL8 signaling promotes a target-associated resistance to 5-FU

**Figure 4:**
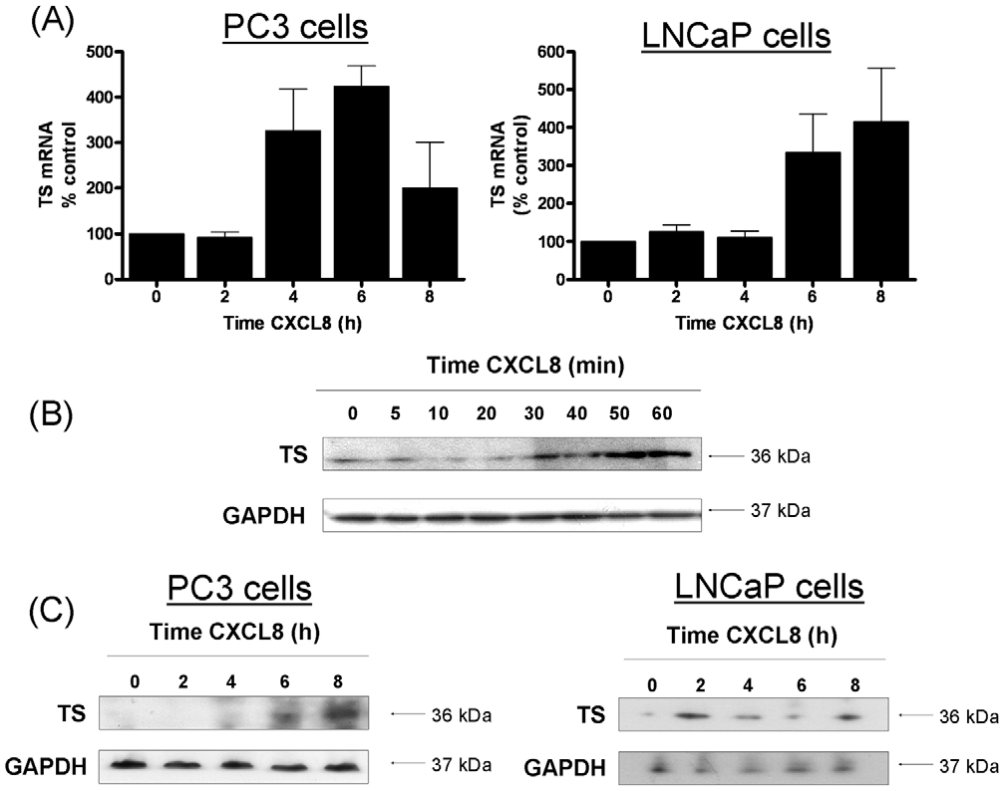
CXCL8 signaling regulates the expression of thymidylate synthase (TS) in PC3 cells. (**A**) Bar graph presenting the time-dependent change in TS transcript levels in PC3 cells (left panel) and LNCaP cells (right panel) as detected by qPCR analysis following treatment with 3 nM rhCXCL8. Expression is presented as a fold-change relative to gene expression levels detected in untreated cells. (**B**) Immunoblot demonstrating the effect of administering 3 nM rhCXCL8 upon the level of TS expression in PC3 cells, detected up to 1 h post-stimulation of the cells. (**C**) Immunoblot that illustrates the level of TS expression detected in PC3 cells (left panel) and LNCaP cells (right panel) up to 8 h post-stimulation of the cells with rhCXCL8. Equal protein loading in each of the representative blots shown in (B) and (C) was confirmed by re-probing of the membranes to detect expression of GAPDH. Statistically significant differences were determined using a Students two-tailed t-test (***, p<0.001).

Thymidylate synthase (TS) expression is a key determinant of 5-FU sensitivity [18], [19]. The increased sensitivity of cells to 5-FU following the repression of CXCL8 signaling prompted us to investigate whether CXCL8 signaling regulated the expression of this enzyme in CaP cells. PC3 cells and LNCaP cells were each stimulated with 3 nM recombinant-human CXCL8 (rh-CXCL8) for a maximum of 8 h. TS gene expression was initially analyzed by quantitative PCR; which confirmed an increase in excess of 4-fold in both cell lines following stimulation with rh-CXCL8 (Figure 4A). Expression of the TS enzyme was also analysed by immunoblotting. Stimulation with rhCXCL8 over a short time-course confirmed an immediate increase in the expression of TS in PC3 cells (Figure 4B). Furthermore, consistent with the increase in mRNA transcript levels, we observed a later response to CXCL8 administration, where TS expression levels were shown to increase at time points out to 8 h in PC3 cells (Figure 4C, left panel) and LNCaP cells (Figure 4C, right panel).

**Figure 5:**
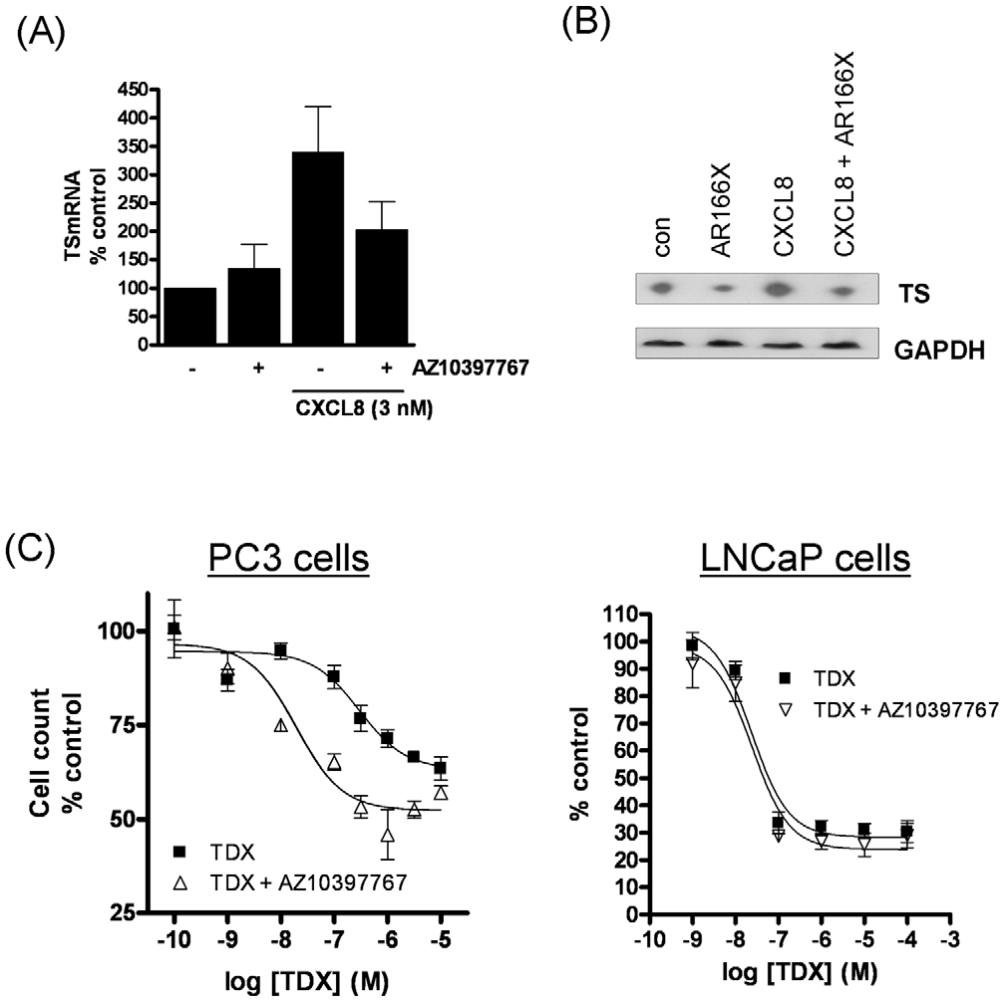
Inhibition of CXCR2 signaling represses TS expression and sensitizes the response to Tomudex. (**A**) Bar graph illustrating the effect of the CXCR2 inhibitor on the CXCL8-promoted increase in TS transcript levels in PC3 cells as detected by qPCR analysis. The inhibitor was added overnight and cells stimulated with 3 nM rhCXCL8 for 8 h. (**B**) A representative immunoblot illustrating the impact of the CXCR2 inhibitor AZ10397767 upon the expression of TS in unstimulated and CXCL8-stimulated conditions. AZ10397767 was added overnight and cells stimulated with 3 nM rhCXCL8 for 8 h. (**C**) Graph illustrating the cell count assay data, determined from the treatment of PC3 cells (left panel) and LNCaP cells (right panel) with increasing concentrations of Tomudex, in the absence or presence of the CXCR2 receptor antagonist, AZ10397767. Cell counts were taken 72 hours post-treatment with the anti-metabolite. Cell count data was analyzed using the non-linear regression function of GraphPad Prism, using a sigmoidal one-site curve fit equation. AZ10397767 was administered at a final concentration of 20 nM in all experiments. Data points shown are the mean ± S.E.M. value of three independent experiments; *p<0.05; **p<0.01.

We next examined how inhibition of CXCR2 signaling affected TS expression. The addition of AZ10397767 to PC3 cells attenuated the CXCL8-induced increase in TS gene transcript levels (Figure 5A) and reduced CXCL8-promoted increases in TS protein (Figure 5B). Given the association of CXCL8 and CXCR2 to the regulation of TS expression, we examined the effect of CXCL8 signaling upon the efficacy of Tomudex (TDX), a potent inhibitor and TS-directed therapeutic. PC3 cells were markedly insensitive to TDX even at high concentrations of the drug. However, as observed with 5-FU, the potency of TDX was markedly increased by the co-administration of the CXCR2 antagonist AZ10397767 (Figure 5C, left panel). Non-linear regression analysis of the cell count data confirmed that the potency (IC30) of TDX was increased by 37-fold, from 1.1 µM to 30 nM in the presence of AZ10397767. The interaction between AZ10397767 and TDX was strongly synergistic, determined using two methods of statistical analysis (RI = 1.4, ANOVA P<0.0001). Our data also shows that the maximal response to TDX is marginally increased in the presence of the CXCR2 antagonist. In contrast, LNCaP cells were much more sensitive to TDX alone, and accordingly, the sensitivity could not be enhanced further by addition of the CXCR2 antagonist (Figure 5C, right panel).

### Inhibition of Bcl-2, a downstream CXCL8-transcriptional target sensitizes CaP cells to 5-FU

**Figure 6:**
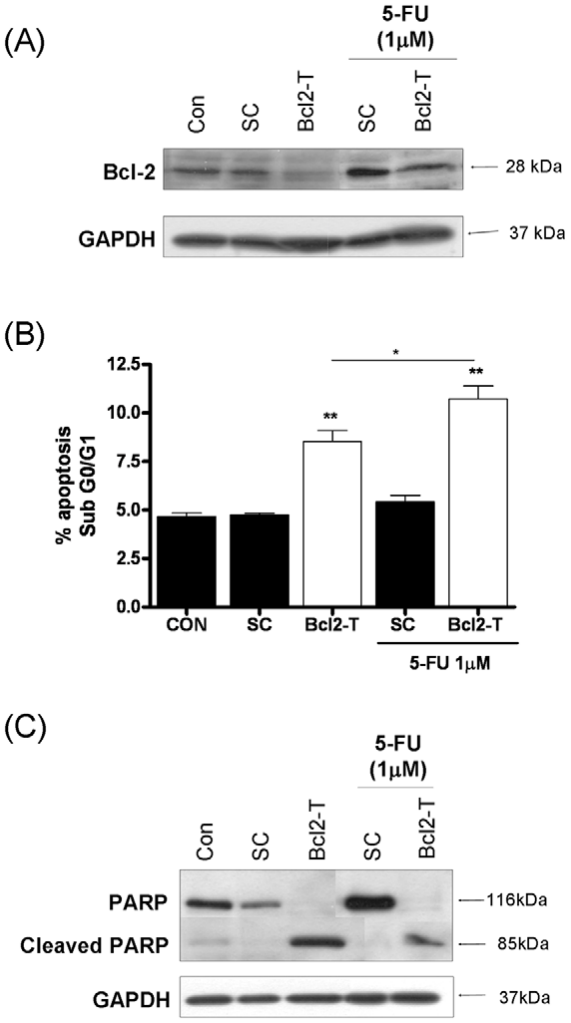
Repression of Bcl-2 potentiates 5-FU-induced apoptosis in metastatic prostate cancer cells. (**A**) Immunoblot presenting the expression level of the anti-apoptotic protein Bcl-2 in PC3 cells transfected with a non-targeting oligonucleotide (Sc) or a gene-targeted oligonucleotide (Bcl2-T). All oligonucleotides were used at a final concentration of 50 nM. Expression of Bcl-2 is presented in the absence or presence of 1 µM 5-FU. (**B**) Bar graph presenting the apoptotic cell population determined by flow cytometry analysis in PC3 cells transfected with a non-targeting oligonucleotide (Sc) or a gene-targeted oligonucleotide (Bcl2-T) at a final concentration of 50 nM, in the absence or presence of 1 µM 5-FU. Statistically-significant differences in the apoptosis levels were determined by conducting two-tailed Student's t-test analysis; *, P<0.05; **, p<0.01. (**C**) Immunoblot presenting the expression and cleavage product of PARP in PC3 cells transfected with a non-targeting oligonucleotide (Sc) or a gene-targeted oligonucleotide (Bcl2-T), used at a final concentration of 50 nM, in the absence or presence of 1 µM 5-FU. Equal protein loading in immunoblots shown in (A) and (C) was confirmed by re-probing the membranes for expression of GAPDH.

CXCL8 signalling increases the expression of several anti-apoptotic genes including Bcl-2 [10]. A small-interfering RNA (SiRNA) strategy was employed to inhibit expression of Bcl-2. Immunoblotting confirmed a high level of endogenous Bcl-2 expression in PC3 cells. Transfection of the cells with a final concentration of a 50 nM Bcl-2-targeted oligonucleotide (Bcl-2-T) reduced but did not eliminate Bcl-2 expression in untreated or 5-FU-treated cell populations. Interestingly, exposure to 5-FU itself was shown to increase Bcl-2 expression in PC3 cells (Figure 6A). Down-regulating Bcl-2 alone had a marked impact in promoting apoptosis in PC3 cells (increasing to 8.5% and P<0.001 relative to scrambled control (SC). While 1 µM 5-FU had limited impact in inducing apoptosis, the combined treatment with 1 µM 5-FU and the Bcl-2 siRNA-oligonucleotides increased the proportion of apoptotic cells to a mean value of 10.7%, (P<0.01 relative to 5-FU treatment alone) (Figure 6B). The increased levels of apoptosis induced by down-regulation of Bcl-2 in the absence and presence of 5-FU was confirmed by the detection of increased PARP cleavage in immunoblotting experiments (Figure 6C).

## Discussion

CRPC is currently an incurable disease. Although the emergence of a new generation of androgen signaling pathway inhibitors (e.g. abiraterone-acetate and MDV3100) for use in CRPC patients will contribute to improved outcomes for many patients, it is clear that a more informed use of conventional chemotherapy agents will be necessary to improve clinical outcomes for patients for whom these new drugs fail to work. 5-FU and its oral analog capecitabine have been reported to be safe as a second-line treatment for metastatic castrate-resistant CaP, with modest responses observed when administered in combination with docetaxel or oxaliplatin in small phase II trials [5], [6]. Increasing the response to second-line chemotherapy is of paramount importance in extending overall survival and the quality-of-life for patients with advanced disease.

In this study we have shown the importance of CXCL8 signaling in underpinning a novel mode of target-associated resistance that decreases the sensitivity of metastatic CaP cells to 5-FU. CXCL8 expression is natively elevated in metastatic or CRPC over normal or benign prostate tissue [7], [8], [20]. Moreover, we have confirmed that exposure to 5-FU potentiates this level of CXCL8 signaling by concurrently inducing CXCL8 secretion and up-regulating CXCR1 and CXCR2 gene expression in metastatic prostate cancer cells. Importantly, inhibition of the intrinsic and drug-induced CXCL8 signaling using a small molecule CXCR2 receptor antagonist resulted in a statistically significant increase in the sensitivity of CRPC cells to 5-FU. While we have exclusively studied the role of CXCL8 in modulating this response, our observations using the CXCR2 inhibitor indicate that stress-induced increases in the additional CXC-chemokines that also activate the CXCR2 receptor including CXCL1 and CXCL5 will also likely contribute to therapeutic resistance.

Elevated expression of TS is a well-established biomarker of decreased 5-FU efficacy in tumours [18], [19]. Administration of either 5-FU or CXCL8 alone was shown to increase TS gene and protein levels. Stimulation with CXCL8 was shown to promote two phases of regulation. We observed a rapid increase in TS expression in response to CXCL8 administration, consistent with our prior studies characterizing the role of this chemokine in regulating protein translation [14]. This was followed by a later-onset increase in protein expression, consistent with the CXCL8-promoted increase in transcription of the TS gene. Administration of a CXCR2 antagonist was shown to attenuate the CXCL8-induced increase in TS gene and protein expression levels. Therefore, our data suggests that CXCL8 signaling manifests a target-associated resistance to 5-FU in CRPC cells by increasing the expression of TS. This conclusion is further supported by our observation that the administration of a CXCR2 antagonist had a profound effect in sensitizing PC3 cells to a direct TS-targeted inhibitor, Tomudex.

In addition, we propose that CXCL8 signaling may additionally contribute a cellular resistance to 5-FU by increasing anti-apoptotic protein expression. Flow cytometry and PARP cleavage assays demonstrated that the protective action of CXCL8 signaling was mediated by enabling the cells to evade chemotherapy-induced apoptosis. We have previously demonstrated that CXCL8 can up-regulate anti-apoptotic expression in CaP cells, including expression of Bcl-2, and that co-administration of AZ10397767 can down-regulate chemotherapy-induced expression of this protein [10], [11]. We provide further evidence that the suppression of Bcl-2 can increase the level of apoptosis in 5-FU-treated cells. Therefore, CXCL8 signalling may also contribute to the resistance of CRPC cells to 5-FU by facilitating an increase in the expression of critical inhibitors of apoptosis.

Our current study reveals a very distinct and drug-specific effect of anti-metabolites on CXCL8 signaling. While 5-FU inhibits TS activity, pemetrexed is a multi-targeted inhibitor with effects upon three folate-dependent enzymes in the thymidine and purine nucleotide synthesis pathways; TS, dihydrofolate reductase (DHFR), and glycinamide ribonucleotide formyltransferase (GARFT) [17]. While 5-FU potentiated CXCL8 secretion and increased CXCL8 receptor expression in PC3 cells, pemetrexed had negligible effects on the expression of CXCL8 or its receptors. Therefore, our observations that the CXCR2 antagonist had no effect upon the sensitivity of cells to pemetrexed may relate to the absence of a pemetrexed-induced increase in CXCL8 signaling and/or reflects that CXCL8 signaling may not regulate the broader spectrum of targets whose activity is modulated by pemetrexed. Although PC3 cells were sensitive and responded favourably to pemetrexed treatment, phase II studies in patients have failed to provide encouragement for the use of this agent as a second line chemotherapy for metastatic CRPC, suggesting that other resistance mechanisms relevant to this drug are at play in this disease setting [21], [22].

CXCL8 signaling is a major pro-inflammatory signal in multiple solid tumors including breast and colorectal cancers [23], [24], diseases in which 5-FU is currently used or recommended as standard-of-care for metastatic disease. Our current observations suggest that the optimal use of 5-FU may only be realized in tumors in which pro-inflammatory signaling, and specifically CXCL8 signaling, is either absent or is repressed during chemotherapy. Moreover, 5-FU is typically used in combination with other chemotherapeutic agents. While platinum-based agents are not routine for the treatment of metastatic CRPC, there is an increasing use of these agents in second-line treatment of metastatic CaP patients who have progressed on docetaxel [6], [25]. Since we have now shown that CXCL8 signaling modulates the sensitivity of metastatic prostate cancer cells to both 5-FU and oxaliplatin, expression of this chemokine could be used as a guide to assist in predicting a patient's response to an oxaliplatin/5-FU combination. Moreover, administration of CXCR2-targeted inhibitors may be especially relevant to exploit as chemo-modulators in any future study of second-line, platinum/5-FU based treatments for metastatic CaP.

In summary, this study adds to a growing literature that documents the relevance of intrinsic and stress/treatment-induced CXC-chemokine signaling in modulating the sensitivity of metastatic CaP cells to chemotherapeutic agents. We have shown that CXCL8 signaling attenuates the efficacy of 5-FU and TDX by a combination of up-regulating anti-apoptotic gene expression and through a novel target-associated resistance mechanism involving increased expression of TS.

## Supporting Information

Figure S1 Graphs illustrating cell count assay data, determined from the treatment of PC3 cells with increasing concentrations of pemetrexed, in the absence or presence of the CXCR2 receptor antagonist, AZ10397767. AZ10397767 was administered at a final concentration of 20 nM. Cell counts were taken 72 hours post-treatment with the anti-metabolite. Cell count data was analyzed using the non-linear regression function of GraphPad Prism, using a sigmoidal one-site curve fit equation. Data points shown are the mean ± S.E.M. value of four independent experiments.
(TIF)

Figure S2 Graph illustrating the percentage distribution of PC3 cells throughout the cell cycle following treatment with 1 µM or 10 µM 5-FU, in the absence or presence of the CXCR2 antagonist AZ10397767. AZ10397767 was administered at a final concentration of 20 nM. Cell cycle profile was analysed 72 hours post-treatment with 5-FU. Data shown is the mean value, determined from three independent experiments.
(TIF)

## References

[1] Attard G, Reid AH, de Bono JS (2010). Abiraterone acetate is well tolerated without the concomitant use of corticosteroids. J Clin Oncol.

[2] de Bono JS, Logothetis CJ, Molina A, Fizazi K, North S (2011). Abiraterone and increased survival in metastatic prostate cancer. N Engl J Med.

[3] Tannock IF, de Wit R, Berry WR, Horti J, Pluzanska A (2004). Docetaxel plus prednisone or mitoxantrone plus prednisone for advanced prostate cancer. N Engl J Med.

[4] Longley DB, Harkin DP, Johnston PG (2003). 5-fluorouracil: mechanisms of action and clinical strategies.. Nature Rev Cancer.

[5] Vaishampayan UN, Marur S, Heilbrun LK, Cher ML, Dickow B (2009). Phase II trial of capecitabine in combination with weekly docetaxel for metastatic castrate resistant prostate cancer. J Urol.

[6] Gasent Blesa JM, Giner Marco V, Giner-Bosch V, Cerezuela Fuentes P, Alberola Candel V (2011). Phase II trial of oxaliplatin and capecitabine after progression to first-line chemotherapy in androgen-independent prostate cancer patients. Am J Clin Oncol.

[7] Murphy C, McGurk M, Pettigrew J, Santinelli A, Mazzucchelli R (2005). Nonapical and cytoplasmic expression of interleukin-8, CXCR1 and CXCR2 correlates with cell proliferation and microvessel density in prostate cancer.. Clin Cancer Res.

[8] Uehara H, Troncoso P, Johnston D, Bucana CD, Dinney C (2005). Expression of interleukin-8 gene in radical prostatectomy specimens is associated with advanced pathologic stage.. Prostate.

[9] Maxwell PJ, Gallagher R, Seaton A, Wilson C, Scullin P (2007). HIF-1 and NF-kappaB-mediated upregulation of CXCR1 and CXCR2 expression promotes survival in hypoxic prostate cancer cells.. Oncogene.

[10] Wilson C, Purcell C, Seaton A, Oladipo O, Maxwell PJ (2008a). Chemotherapy-induced CXC-chemokine/CXC-chemokine receptor signalling in metastatic prostate cancer cells confers resistance to oxaliplatin through potentiation of nuclear factor-kappaB transcription and evasion of apoptosis. J Pharm Exp Ther.

[11] Wilson C, Wilson T, Johnston PG, Longley DB, Waugh DJ (2008b). Interleukin-8 signaling attenuates TRAIL- and chemotherapy-induced apoptosis through transcriptional regulation of c-FLIP in prostate cancer cells. Mol Cancer Ther.

[12] Araki S, Omori Y, Lyn D, Singh RK, Meinbach DM (2007). Interleukin-8 is a molecular determinant of androgen-independence and progression in prostate cancer. Cancer Res.

[13] Seaton A, Scullin P, Maxwell PJ, Wilson C, Pettigrew J (2008). Interleukin-8 signaling promotes androgen-independent proliferation of prostate cancer cells via induction of androgen receptor expression and activation. Carcinogenesis..

[14] MacManus CF, Pettigrew J, Seaton A, Wilson C, Maxwell PJ (2007). Interleukin-8 signaling promotes translational regulation of cyclin D1 in androgen-independent prostate cancer cells.. Mol Cancer Res.

[15] Seaton A, Maxwell PJ, Hill A, Gallagher R, Pettigrew J (2009). Inhibition of constitutive and CXC-chemokine-induced NF-kappaB activity potentiates ansamycin-based Hsp90-inhibitor cytotoxicity in castrate-resistant prostate cancer cells.. Br J Cancer.

[16] Kaighn ME, Narayan KS, Ohnuki Y, Lechner JF, Jones LW (1979). Establishment and characterization of a human prostate adenocarcinoma cell line (PC-3).. Invest Urol.

[17] Adjei AA (2004). Pemetrexed (ALIMTA): a novel multitargeted antineoplastic agent.. Clin Cancer Res.

[18] Johnston PG, Lenz HJ, Leichman CG, Danenberg KD, Allegra CJ (1995). Thymidylate synthase gene and protein expression correlate and are associated with response to 5-fluorouracil in human colorectal and gastric tumors. Cancer Res.

[19] Salonga D, Danenberg KD, Johnson M, Metzger R, Groshen S (2000). Colorectal tumors responding to 5-fluorouracil have low gene expression levels of dihydropyrimidine dehydrogenase, thymidylate synthase, and thymidine phosphorylase. Clin Cancer Res.

[20] Veltri RW, Miller MC, Partin AW, Poole EC, O'Dowd GJ (1999). Interleukin-8 serum levels in patients with benign prostatic hyperplasia and prostate cancer.. Cancer.

[21] Hahn NM, Zon RT, Yu M, Ademuyiwa FO, Jones T (2009). A phase II study of pemetrexed as second-line chemotherapy for the treatment of metastatic castrate-resistant prostate cancer (CRPC)- Hoosier Oncology Group GU03-67.. Ann Oncol.

[22] Caffo O, Fratino L, Barbieri R, Perin A, Martini T (2011). Pemetrexed as second-line chemotherapy for castrate-resistant prostate cancer after docetaxel failure: results from a phase II *study.*. Urol Oncol, epub ahead of print Jul.

[23] Bendre MS, Gaddy-Kurten D, Mon-Foote T, Akel NS, Skinner RA (2002). Expression of interleukin 8 and not parathyroid hormone-related protein by human breast cancer cells correlates with bone metastasis in vivo. Cancer Res..

[24] Ning Y, Manegold PC, Hong YK, Zhang W, Pohl A (2011). Interleukin-8 is associated with proliferation, migration, angiogenesis and chemosensitivity.. Int J Cancer.

[25] Droz JP, Muracciole X, Mottet N, Ould Kaci M, Vannetzel JM (2003). Phase II study of oxaliplatin versus oxaliplatin combined with infusional 5-fluorouracil in hormone refractory metastatic prostate cancer patients. Ann Oncol.

