# Supplementary figures and images for: Constitutive and Treatment-Induced CXCL8-Signalling Selectively Modulates the Efficacy of Anti-Metabolite Therapeutics in Metastatic Prostate Cancer
Source: PLoS One. 2012 May 9;7(5):e36545. doi: 10.1371/journal.pone.0036545 (PMC3348872; doi:10.1371/journal.pone.0036545)

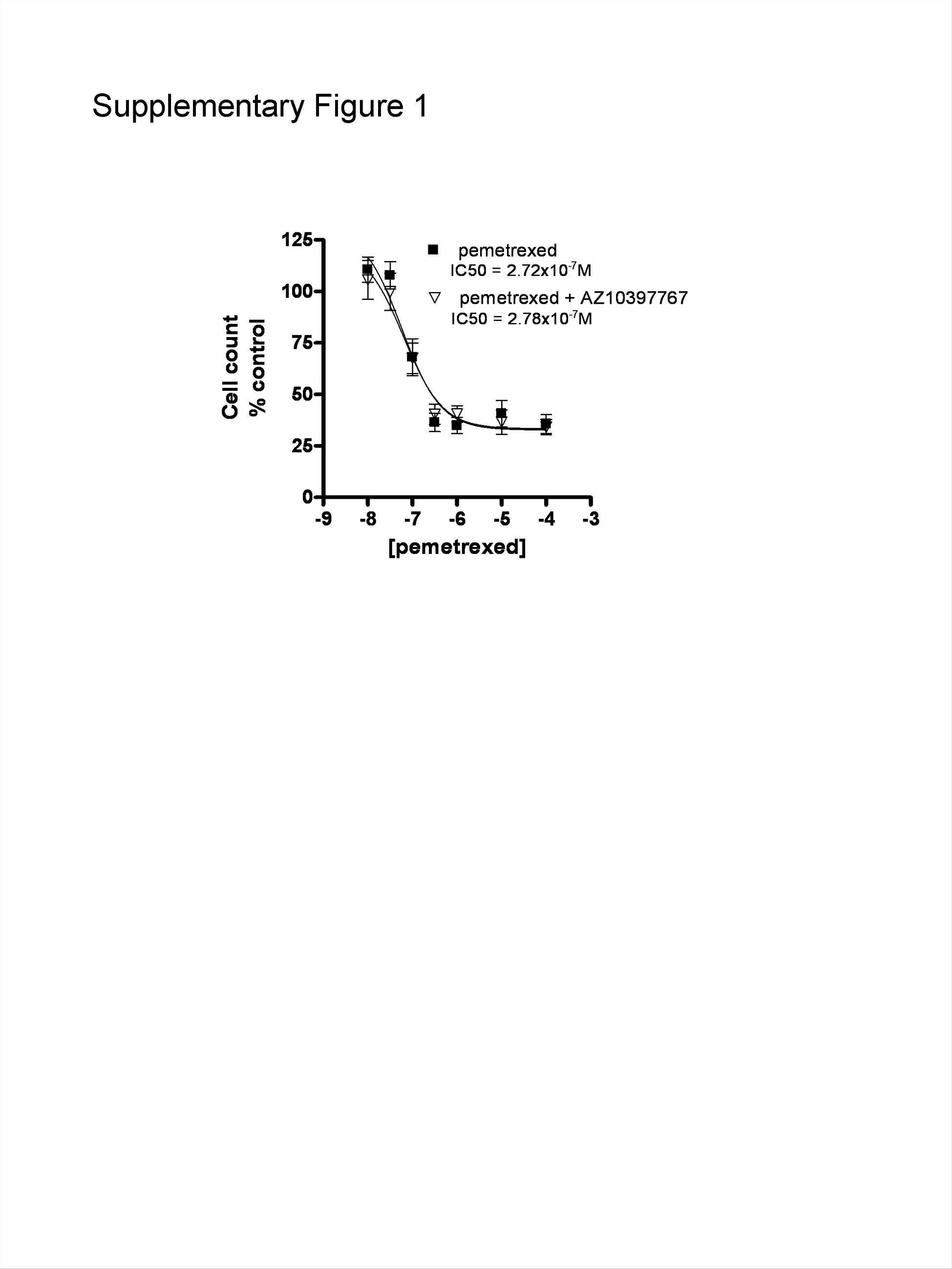

Supplement: Figure S1 — Graphs illustrating cell count assay data, determined from the treatment of PC3 cells with increasing concentrations of pemetrexed, in the absence or presence of the CXCR2 receptor antagonist, AZ10397767. AZ10397767 was administered at a final concentration of 20 nM. Cell counts were taken 72 hours post-treatment with the anti-metabolite. Cell count data was analyzed using the non-linear regression function of GraphPad Prism, using a sigmoidal one-site curve fit equation. Data points shown are the mean ± S.E.M. value of four independent experiments. (TIF) [file pone.0036545.s001.tif]

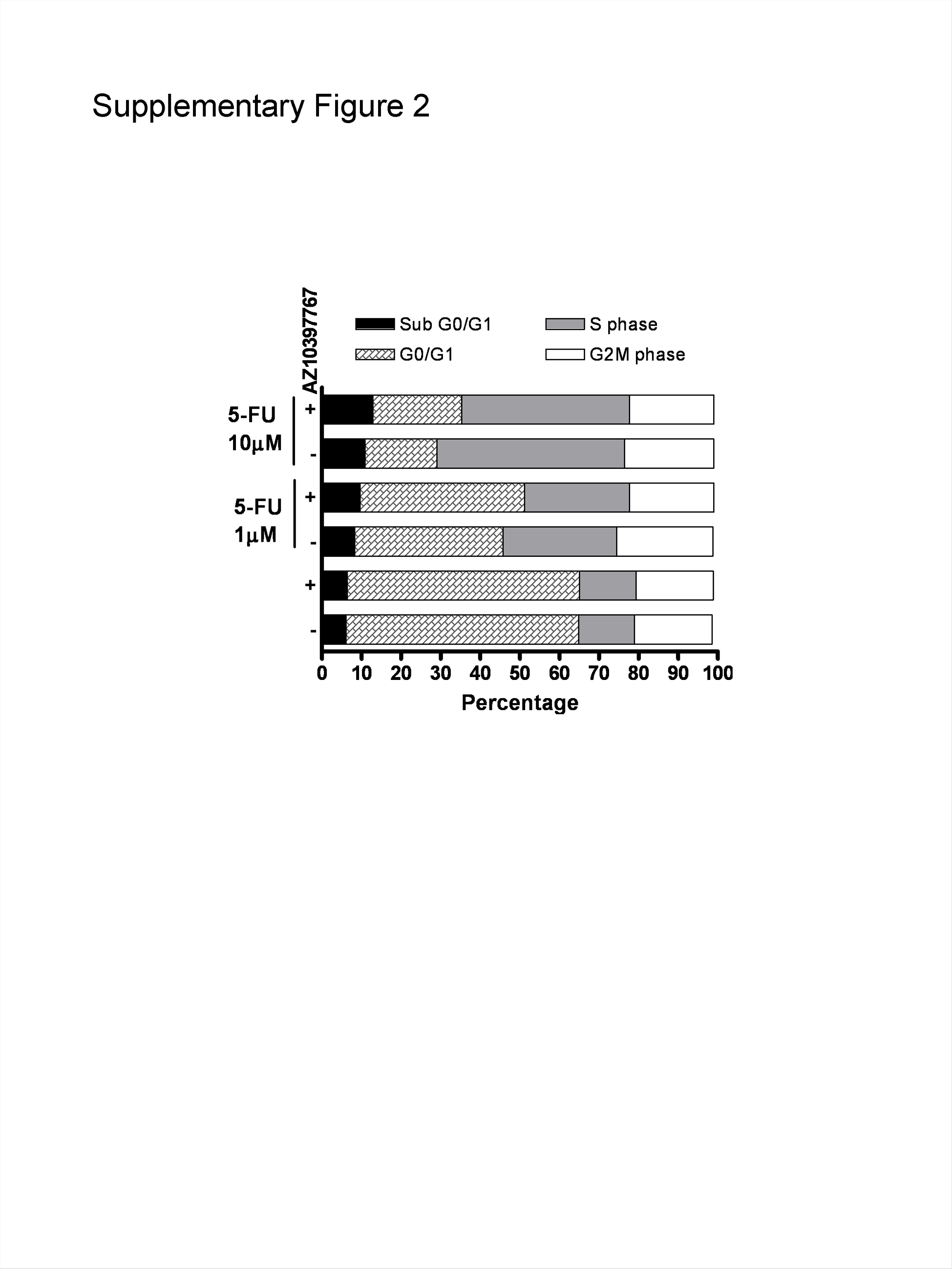

Supplement: Figure S2 — Graph illustrating the percentage distribution of PC3 cells throughout the cell cycle following treatment with 1 µM or 10 µM 5-FU, in the absence or presence of the CXCR2 antagonist AZ10397767. AZ10397767 was administered at a final concentration of 20 nM. Cell cycle profile was analysed 72 hours post-treatment with 5-FU. Data shown is the mean value, determined from three independent experiments. (TIF) [file pone.0036545.s002.tif]
